# Decreased Capacity for Vitamin A Storage in Hepatic Stellate Cells for Arctic Animals

**DOI:** 10.1186/1476-5926-2-S1-S18

**Published:** 2004-01-14

**Authors:** Haruki Senoo, Kenjiro Wake, Heidi L Wold, Nobuyo Higashi, Katsuyuki Imai, Jan Øivind Moskaug, Naosuke Kojima, Mitsutaka Miura, Takeya Sato, Mitsuru Sato, Norbert Roos, Trond Berg, Kaare R Norum, Rune Blomhoff

**Affiliations:** 1 Department of Anatomy, Akita University School of Medicine, Akita, 010-8543, Japan; 2 Liver Research Unit, Minophagen Pharmaceutical Co. Ltd., Tokyo, 160-0004, Japan; 3 Institute for Nutrition Research, Faculty of Medicine, University of Oslo, Oslo, Norway; 4 Department of Biology, University of Oslo, Oslo, Norway

## Introduction

Under physiological conditions, hepatic stellate cells store 80 % of the total vitamin A in the whole body as retinyl palmitate in lipid droplets in the cytoplasm, and regulate both transport and storage of vitamin A [1-3].

It has been demonstrated that animal or human individuals exposed to drugs such as methadone, prednisone and phenobarbital, antiepileptics [4], and xenobiotics like the environmental contaminants DDT, PCD and dioxins have dramatic changes in their retinoid metabolism and function.

Recent data demonstrate that top predators among Svalbard mammals and birds like polar bear, arctic fox and glaucous gull accumulate relatively large amounts of persistent organic pollutants [5]. Since it has been reported that vitamin A accumulates at near toxic doses in some arctic predators [6] and recent data suggest that PCB and DDT may reduce the threshold for vitamin A-toxicity [7], an increasing accumulation of persistent organic pollutants might eventually precipitate vitamin A-toxicity in these animals. To elucidate the possibility of vitamin A-related toxicity in arctic predators, we have performed a systematic characterization of the hepatic vitamin A-storage, which is the best index of the vitamin A-status, in mammals of the Svalbard archipelago.

## Methods

After getting permission to hunt the animals from the district governor of Svalbard, 11 arctic foxes and 14 bearded seals, were caught in the Svalbard archipelago near Longyearbyen (78Ø N, 15Ø E) in the period from August 1996 to September 2001. Three polar bears were shot in self-defense at Svalbard February and August 1998 in Ny Ølesund and Hornsund. Distribution and content of vitamin A in livers and other organs were analyzed by morphological methods such as transmission electron microscopy, fluorescence microscopy for detection of autofluorescence of vitamin A and gold chloride staining [8] and high-performance liquid chromatography.

## Results and Discussion

The amounts of vitamin A stored in livers of arctic animal are shown in Table 1. The median values are presented due to the relatively large individual variations. These values are much higher than all other arctic animals studied as well as their genetically related continental top predators (data not shown). The values are also high compared to normal human values and experimental animals like mouse and rat (200Ø600 nmol per gram) [9].

**Table 1:** Total retinol concentration in liver and kidney from rats and arctic animals.

	Total retinol concentration^a^		
	
**Species**	Liver (nmol/g)	Kidney (nmol/g)	Kidney concentration as percent of liver concentration
Control rats (n = 3)^b^	1106	9	0.81
Vitamin A-fed rats (n = 3)^b^	16877	224	1.33
**Arctic animals:**			
Polar bear (n = 3)	18279	102	0.56
Arctic fox (n = 8)	18641	1623	8.71
Bearded seal (n = 10)	4652	39	0.84

^a ^Total retinol content (i.e. the sum of retinol and retinyl esters) is presented as mean for rats and median for arctic animals. ^b ^These results have been published in a previous study [9].

The concentration of total retinol in kidney is normally less that 1% of the concentration in liver. However, following an intake of excessive amounts of vitamin A or after experimental treatment with xenobiotics like HCB, TCDD and PCB, the concentration in kidneys may increase several-fold [10,11]. Kidney total retinol may therefore be used as a biomarker for vitamin A-related toxicity or excess. When we measured the kidney concentration of total retinol in the top arctic predators we observed that polar bear and bearded seal had kidney levels below 1% of their liver value (Table 1). Arctic fox, however, had kidney levels of about 9% of the liver values (Table 1).

Strong autofluorescence and gold chloride staining were present in hepatic stellate cells of polar bear, arctic fox, and bearded seal (data not shown). The distribution of stored vitamin A in the arctic animals was essentially the same as that published previously in normal rat and human liver [2,3]. In livers of arctic fox, polar bear, and beared seal, stellate cells stored one or two large lipid droplets in their cytoplasm (data not shown). In the kidney of arctic foxes and bearded seals, interstitial cells (renal stellate cells) stored vitamin A-lipid droplets (data not shown).

Increased kidney concentrations of total retinol in arctic fox most likely are a sign of pollutant-induced vitamin A-toxicity. It is interesting to note that the highest accumulation of organic pollutants in arctic animals occur in the arctic fox [12]. Relative decrease of liver concentration of total retinol in arctic fox might be due to the decreased capacity for storage of vitamin A in hepatic stellate cells.
